# Characterization of sample preparation methods of NIH/3T3 fibroblasts for ToF-SIMS analysis

**DOI:** 10.1186/1559-4106-8-15

**Published:** 2013-07-05

**Authors:** Michael A Robinson, David G Castner

**Affiliations:** 1 National ESCA and Surface Analysis Center for Biomedical Problems, University of Washington 98195 Seattle, WA, USA; 2 Department of Chemical Engineering, University of Washington, 98195, Seattle, WA, USA; 3 Department of Bioengineering, University of Washington, 98195, Seattle, WA, USA

**Keywords:** ToF-SIMS, Cells, Depth profile, Sample preparation

## Abstract

The information that is obtained from single cells during time-of-flight secondary ion mass spectrometry (ToF-SIMS) analysis is influenced by the method that was used to prepare the cells. The removal of extracellular media before analysis is necessary, but the rinsing technique should not damage the plasma membrane of the cell. The presence of intracellular salts reduced the secondary ion yield an average of 2.6-fold during Bi_3_^+^/C_60_^++^ depth profiles. Chemical fixation followed by rinsing removed a majority of the intracellular salts, “recovering” the positive secondary ion yields. The formaldehyde-fixation process removed a majority of the intracellular Cl^-^, but other key anions were not removed in significant amounts. The data presented here is consistent the anion neutralization mechanism largely responsible for the lower ion yields. All of the organic secondary ions that were detected in the freeze-dried cells were also detected in the formaldehyde-fixed cells, suggesting that the fixation process did not remove any molecular species to an extent that is detectable by ToF-SIMS. Compared to freeze dried cells, well preserved, frozen-hydrated cells showed little increase, or a decreased yield, for most low mass ions, but an increased yield for larger mass fragments. This is consistent with a reduced damage cross section at cryogenic analysis temperatures, although proton donation from water and reduction the salt effects in the presence of water likely also play roles. Numerous ions detected from the frozen-hydrated cells were not detected from the freeze dried cells, however many of these ions were attributed to chemical combinations of water, salts and the ammonium acetate rinsing solution.

## Background

Time-of-flight secondary ion mass spectrometry (ToF-SIMS) is a powerful tool that has been used to explore a wide range of biologically relevant samples including: cells and tissues [1–4], lipids [5], proteins on surfaces [6, 7], DNA [8, 9], drug eluting stents [10, 11], explanted biomaterials [12] and decellularized matrix [13]. The unique abilities of ToF-SIMS to acquire a mass spectrum with high mass resolution, as well as produce chemical maps with submicron spatial resolution [14] provides an effective method to probe biological cells and tissues. These strengths, along with the capacity to sputter etch organic material [15, 16], may enable the 3D visualization of sub-cellular features including drug, metabolite or nanoparticle behavior within single cells. There is a continuing need to characterize how diverse sample preparation methods influence the information that is acquired from biological cells [17].

**Figure 1:**
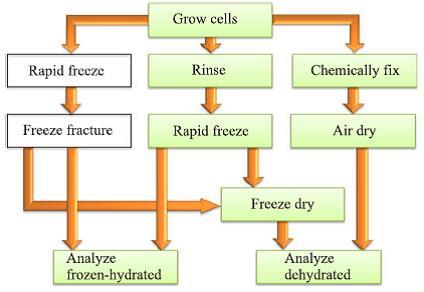
**A schematic of several of the methods used to prepare biological cells for ToF-SIMS analysis.** Cells can either be analyzed following dehydration, or can be analyzed frozen-hydrated with incorporated water still present. Cells can be dried either by chemical fixation and air drying, or plunge-frozen and freeze-dried. Additionally, frozen cells can either be analyzed whole, or following freeze-fracture. The green boxes represent methods that were evaluated in this work.

The methods to prepare cells for SIMS analysis can be organized in several ways. A schematic that outlines the most common techniques is shown in Figure 1. They can be separated into one of two fundamental categories: either the cells are dehydrated prior to analysis, or the intracellular water is conserved and the cells are analyzed frozen-hydrated. The methods may also be organized by the mechanism that removes the culture media from a cells’ surface before analysis. Either the media is removed with an isotonic rinsing solution, or the cells are freeze-fractured, removing a portion of the cell and all of the media above it, creating a pristine surface for analysis [18, 19].

One of the most common constituents in biological specimens are salts, particularly Na^+^, K^+^ and Cl^-^, which greatly lowered the organic SI yields in rat brain tissue and DPPC films [20], as well as bovine serum albumin and polypeptide films [21]. SI yields were “recovered” by rinsing these samples, which removed most of the salts. On single cells, several rinsing solutions were compared, and it was determined that an isotonic solution (150 mM) of ammonium acetate (AA) best removed extracellular growth medium salts and best preserved cell morphology [22]. Ammonium formate (AF) has worked similarly on cells [23] and tissues [21, 24, 25]. Interference reflection microscopy determined the volume change of cells immersed in an AF rinsing solution. At 1 minute of immersion time there was a roughly 1.4× volume increase and at 16 minutes the cells had experienced a 2× volume increase. The swelling amount was not sufficient to rupture the plasma membrane and many cells were viable after a 16 minute immersion [24].

Breitenstein *et al*. used gluteraldehyde-fixation to image single NRK cells in 3D and argued the merits of chemical fixation as a method to prepare cells for ToF-SIMS analysis [26, 27]. The key conclusions included gluteraldehyde-fixation did not cause significant shrinkage of the cell, that cellular compartments remained intact following fixation, and that the distribution of most molecular chemical components (proteins, DNA, lipids) was conserved.

The effects of cryofixation and freeze-drying, as well as various chemical fixations on the morphology and SI yields from the plasma membrane of human fibroblasts were compared by Malm *et al*. [24]. While the cells’ extracellular fine structure was better preserved using chemical fixation, the native intracellular K^+^/Na^+^ ratio was not maintained. The permeability of the plasma membrane to mobile ions was also utilized in early ion microprobe experiments which tested practical ion yields [28]. Intracellular K^+^/Na^+^ ratios have been used as one indicator to determine the quality of cell preservation. Popularized in the SIMS community by Chandra *et al*., this ratio has been used to identify healthy cells from damaged ones [18, 29, 30], where a healthy cell has a high K^+^/Na^+^ ratio. Membrane damage, caused by either the freezing or drying processes resulted in increased permeability to diffusible ions and the loss of this natural gradient. Many of these issues were discussed by Clerc *et al*. with an emphasis on mapping intracellular drugs and nuclear medicine compounds [31]. A major assertion was that cryofixation methodologies are required when examining highly mobile ions and chemical fixation should only be employed when studying molecules fixed to cell structures.

The investigation of frozen-hydrated (FH) cells with ToF-SIMS may be beneficial for several reasons. First, it allows a cell to be studied in its most native state. Rapid freezing provides a “snapshot” of the cell at a given instant, arresting all biological activity on a millisecond time scale [32]. Dehydration may cause cells to shrink or crack, may redistribute membrane lipids and/or cause plasma membranes to rupture [22, 24, 33]. However shrinking was not reported [26] and membrane lipid distributions were preserved using gluteraldehyde-fixation [34]. At cryogenic temperatures, depth resolution and SI yield were improved in depth profiling polymers, partly by reducing sputter-induced topography. [35, 36]. Several studies have reported increased SI yields in FH analyses, and suggested that the increased yield resulted from proton donation from incorporated water molecules to neutral species [37, 38]. Incorporated water during FH analysis has also alleviated the reduction of SI yields caused by KCl-doped arginine films, restoring yields to those that were acquired on undoped films [20]. Cryogenic analysis reduced the damage cross section of primary ions, which improved the SI yields of larger molecular weight ions in polypeptide films [37].

When cells are analyzed FH with ToF-SIMS, a majority of surface water must be removed due to the 1–2 nm surface sensitivity it provides. A practical method to remove this surface water is *in situ* sublimation [38–40]. This is generally achieved by slowly warming the sample stage from cryogenic temperature to −80°C, effectively freeze-drying the sample for a short period of time. With FH cell analysis, sublimation is often a necessary step to remove any rinsing solution left over from the removal of the culture media.

Instrumentation developments that specifically addressed improving FH SIMS experiments include Ionoptika’s J105, which allows for the *in*-*situ* freeze fracturing of a frozen cells under vacuum [41]. A cryomicrotome was attached to a ToF-SIMS instrument to serial section frozen tissue *in situ* and analyze the fresh face of sequential tissue slices [42]. Also, recently an *in*-*situ* freeze fracture device was adapted from the J105 freeze fracture system to work on an ION-TOF IV cold stage [43].

As summarized above, there is significant interest and challenges in using different sample preparation procedures for ToF-SIMS imaging of biological cells. The current study provides a comprehensive comparison of the mass spectral information that was obtained from formaldehyde-fixed cells, cryofixed and dehydrated cells, and frozen-hydrated cells. The surface spectra were obtained from cells that were prepared using each preparation method and examined. The secondary ion yields from depth profiles of the chemically fixed cells were compared to freeze-dried cells. A second SI yield comparison was made between freeze-dried cells and frozen-hydrated cells. All organic ions in all depth profiles were investigated. While there have been studies in the past that have examined SI yields as a function of cell preparation method [24, 33], this is the most complete analysis to date. Additionally, several peaks from the frozen-hydrated analyses were identified that were not detected from the dried cells. This work offers additional insight into which are the primary mechanisms of SI yield enhancement and degradation during ToF-SIMS depth profiling of biological cells.

## Methods

### Cell seeding

NIH/3T3 fibroblasts were seeded onto 1 cm × 1 cm silicon chips [44] at densities between 40,000-100,000 cells and grown for 24–48 hours in Eagle’s Dubelco’s Modified Essential Medium (Invitrogen, San Diego, CA) supplemented with 10% fetal bovine serum (Thermo Scientific, Erie, PA) and 1% antibiotic/antimycotic (Invitrogen, San Diego, CA). Prior to cell seeding the silicon chips were cleaned with 2x sequential five-minute sonications in dichloromethane, acetone and methanol and stored in a laminar hood until cell seeding.

### Ammonium acetate rinsing solution

Ammonium acetate (AA) (Sigma, St. Louis, MO) was dissolved in 18 MΩ water to form a 150 mM solution, and was brought to pH 7.4 with 1 M ammonium hydroxide [22].

### Rinsing, plunge freezing and freeze-drying of cells

Cells on silicon chips were gently rinsed in 150 mM AA for 30 seconds by slowly dipping the silicon chip in the solution followed by minimal movement of the sample in the rinsing solution. Excess liquid was removed by touching the edges of the chip with a Kimwipe. The sample was then rapidly submerged in liquid ethane (produced by leaking ethane gas into a liquid nitrogen cooled plastic beaker) and quickly transferred to liquid nitrogen (LN_2_). While under LN_2_, the chips were placed into small glass test tubes, and the tops were covered with aluminum foil with a small hole. These test tubes were placed into a pre-cooled freeze-dry flask (−80°C freezer overnight), while the chips were still submerged in LN_2_. The flask was attached to a manifold freeze drier (SP Scientific, Warminster, PA) and a vacuum was established. The samples dried overnight, and then were immediately placed into the ToF-SIMS instrument for analysis.

### Chemical fixation

Cells on silicon chips were rinsed briefly in the AA solution and placed into a 4% formaldehyde (Thermo Scientific, Erie, PA) in PBS buffer (EDS Chemicals) solution at room temperature for 30 minutes. The samples were removed and rinsed for 60 seconds in water. Excess liquid was removed from the samples by touching the edges with a Kimwipe, and then they were air dried overnight in a laminar flow hood. The samples were analyzed the following morning.

### Frozen-hydrated: preparation of cells and analysis

Samples were prepared in a manner similar to that described in Piwowar *et al*. [45]. Cells on silicon chips were rinsed and plunge frozen as described above. Prior to freezing, it is important to reduce the thickness of the liquid layer to a minimum in order to ensure the best freezing results. After cryofixation in liquid ethane, the samples were transferred to LN_2_ until placement onto the ToF-SIMS cold stage. The cold stage was pre-cooled to −160°C at 10^-7^ mbar in the loading chamber, then vented to atmosphere to allow for the placement of the sample. The sample was rapidly transferred from under liquid nitrogen onto the cold stage, where it was held onto the stage by a clip. The cold finger was immediately brought into contact with the cold stage and the loading chamber was immediately pumped down to 5×10^-7^ mbar. The maximum temperature of the cold stage was −85°C during the entire process, as measured by a thermocouple mounted to the surface of the stage. After the loading chamber was evacuated the sample stage was cooled to −130°C before warming at a rate of 5°C/min to −80°C [38]. The stage was held at −80°C for 30 minutes to sublimate the excess water from the surface of the cells. After the sublimation, the stage was cooled to −160°C and transferred into the analysis chamber. All analyses were performed with the stage temperature at −130°C. Cells maintained a high K^+^/Na^+^ ratio, as shown in Additional file 1: Figure S1.

### ToF-SIMS

Positive and negative secondary ion spectra were collected with an ION-TOF TOF SIMS 5–100 instrument (ION-TOF, Münster, Germany), using a pulsed 25 keV Bi_3_^+^ primary ion beam. Samples were sputtered using a 20 keV C_60_^++^ beam. The Bi and C_60_ beams were oriented 45° to the surface normal. Depth profiles were acquired using the high mass resolution mode (m/Δm = 7000 at m/z 27) in the interlaced mode (one analysis scan per sputter cycle) with a Bi_3_^+^ current of 0.15 pA and an analysis area of 500 × 500 μm^2^. The C_60_^++^ current was kept between 0.3-0.35 nA and a sputter area of 700 × 700 μm^2^ was used for all depth profiles. All depth profiles were acquired for 1000 seconds, one shot/pixel and contained 128×128 pixels. Positive ion spectra were mass calibrated using the CH_3_^+^, C_2_H_3_^+^, C_3_H_5_^+^, C_3_H_3_O^+^, and C_7_H_7_^+^ peaks. Negative ion spectra were mass calibrated using the CH^-^, OH^-^, PO_2_^-^, and PO_3_^-^ peaks. Secondary ions were collected over a range of 0–860 m/z. Low energy electrons were flooded onto the sample to compensate for charge buildup on the surface. Some data was collected in the .RAW format using the IONTOF Version 4 software and subsequently converted into the .itm raw data format (IONTOF Surface Lab 6). All data was analyzed using the IONTOF Surface Lab 6 software.

Regions of interest (ROI’s) for the cells were chosen in all analyses by thresholding the “totalcounts” image. The signal from the cells was significantly higher than from the substrate. Regions were chosen so that a minimum of substrate would be analyzed during the depth profiles. The SI intensities from the depth profiles were normalized by the primary ion dose to allow direct comparison. Peak lists were created by overlaying representative spectra from each of the preparation methods and using manual peak selection. Known substrate, salt and salt adduct peaks were excluded from the peak lists.

## Results and discussion

The purpose of these experiments was to examine how various methods of sample preparation affected the chemical information obtained from single cells, with an emphasis on SI yields from depth profiles. This work compares cells that were prepared by formaldehyde-fixation (FF), plunge freezing and freeze-drying (FD), and plunge freezing with frozen-hydrated analysis (FH). This is important for 3D ToF-SIMS imaging, where SI intensities of organic species often quickly decrease due to sputtering induced damage [46].

### Proper rinsing of cells

**Figure 2:**
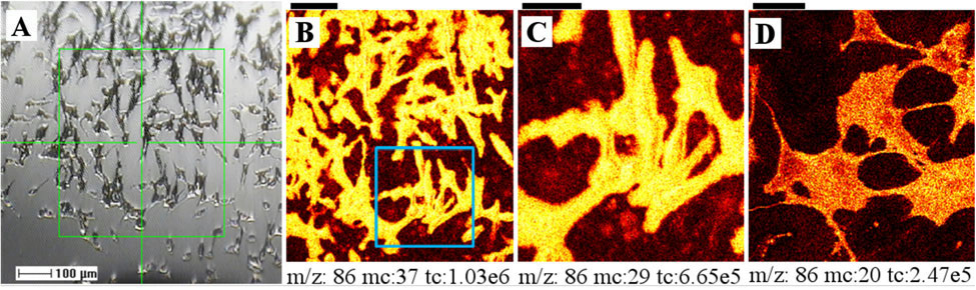
**Excessively harsh rinsing can damage a cell’s plasma membrane.** NIH/3 T3 fibroblasts adhered to a silicon chip were rinsed vigorously for 30 seconds in a 150 mM ammonium acetate solution **(A)** Optical image from the analysis chamber of a group of NIH/3 T3 fibroblasts. **(B)** Ion image of m/z 86^+^, corresponding to C_5_H_12_N^+^, a characteristic phosphocholine head group fragment. Scale bar is 100 μm. **(C)** Higher magnification view of the area inside the box shown in **(B)**. The lipid bilayer looks to have ruptured causing some lipid to spread into the area around the cells. Scale bar is 50 μm. **(D)** An image of the m/z 86^+^ fragment of a group of cells on a separate sample that was more gently rinsed. There is no such damage to the cells’ outer membrane. Scale bar is 50 μm.

The removal of excess media from the surface of cells prior to analysis is an important step in the preparation protocol for ToF-SIMS analysis [22, 24]. An improper rinsing technique can stress the plasma membrane such that it is damaged, as depicted in Figure 2. In an effort to completely remove extracellular media salts from the surface of the cells prior to cryofixation, the sample was immersed in a rapidly stirred AA solution. The cells were submerged for 30 seconds, plunge frozen and freeze dried. An optical image from the analytical chamber of the ToF-SIMS instrument is shown in Figure 2A, where a group of fibroblasts is displayed. A high spatial resolution image of the characteristic phosphocholine (PC) head group fragment at m/z 86 (C_5_H_12_N^+^) is shown in 2B, and 2C is a zoomed-in area of a subset of cells from 2B (blue square). Comparing the lipid distribution from 2C to the optical image from 2A, the plasma membranes appear “smeared”. Shear stress on the membrane from the rapidly moving fluid may have caused this disruption. In Figure 2D a cluster of cells from a separate sample are shown that were gently dipped into a stagnant rinsing solution for 30 seconds and then plunge frozen and freeze dried. No smearing of the cell membrane is visible and sufficient salts have been removed to acquire a meaningful spectrum. Thus, to rinse cells without causing plasma membrane smearing, samples should be gently dipped into a rinsing solution for 30–60 seconds. Vigorous stirring should be avoided.

### Spectra from cell surfaces

**Figure 3:**
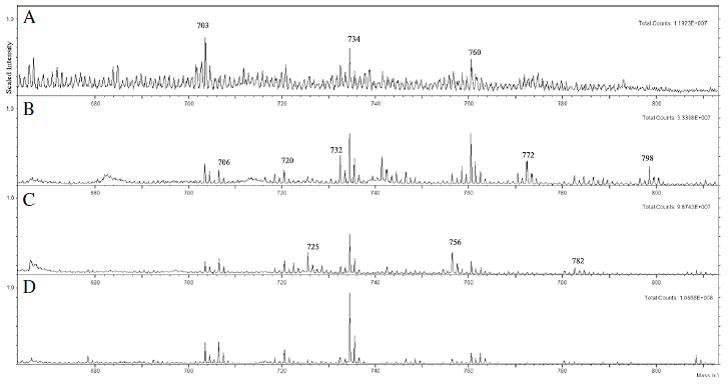
**Spectra from the surface of cells that were analyzed (A) frozen-hydrated, (B) freeze-dried, (C) formaldehyde-fixed followed by a light rinse and (D) formaldehyde-fixed followed by a heavy rinse.** Each spectrum was normalized to the total ion intensity, and all spectra were plotted with the same y-scale. All spectra were acquired with a 250 μm × 250 μm analysis area and to a primary ion dose density of 1.0 × 10^12^ ions/cm^2^. Peaks assignments are given in the text.

Figure 3 shows the positive SI spectra between m/z 660–810, collected from the surface of NIH/3T3 fibroblasts that were analyzed after the following preparation methods: A) FH, B) FD, C) FF with a light rinse after fixation and D) FF with a heavy rinse after fixation. Each spectrum was normalized to the total counts detected and plotted on the same y-scale. The ions at m/z 703, 732, 734 and 760 were detected in the spectra from cells using each of the preparation methods. These ions were identified as sphingomyelin SM(34:1) [47], glycerophosphocholine PC(32:1), PC(32:0) [48], and PC(34:1) [49]. The peak at m/z 762.2 was identified as PC(34:0). An unidentified peak at m/z 720.5 was present in each of the spectra. An unidentified peak at m/z 706.5 was present in all but the FH spectrum. Th**e** K^+^ adducts of SM(34:1), PC(32:1), PC(32:0) and PC (34:1) were detected at m/z 741, 770, 772 and 798, whereas the Na^+^ adducts of these lipids were detected at m/z 725, 754, 756 and 782, respectively [25]. The K^+^ adducts were not detected in high levels in either of the FF samples, consistent with previous experiments that showed a majority of K^+^ was removed during the fixation process [24]. The FF sample that was not rinsed well after fixation had the highest intensity of Na^+^ adduct ions, as expected since Na^+^ is a major component of the PBS fixation buffer.

The FH and FD spectra were more similar to one another than to the FF spectra, however all four spectra contained the same major peaks. The largest differences between the spectra from the FD (3B) and FF (3D) cells were the lowered intensity of PC(34:1) and PC(32:1), and the increased intensity of PC(32:0) in the FF spectrum. This is consistent with previous results where the normalized intensity of PC(34:1) was lower in gluteraldehyde-fixed cells compared to cryofixed and FD cells [24]. The SM(34:1) ion had a comparable intensity to the PC(32:0) and PC(34:1) peaks in the FH spectrum, whereas in all three spectra of dried cells the SM(34:1) intensity was lower than the PC peaks. These differences in the spectra may have been caused by the increased disorganization of the plasma membrane as the cells shifted from fully hydrated (most native), to freeze dried, to formaldehyde-fixed (least native).

The spectrum from the FH cells was similar to that of the FD cells likely because the FH cells were subjected to a −80°C environment for a short time to sublime excess surface water. Although a thin layer of water was redeposited onto the surface after this sublimation period (Additional file 2: Figure S2 C), this would likely not reverse any re-organization of the membrane caused by the initial freeze-drying, although it likely would increase ion yields. An ideal preparation to study the plasma membrane would stop the sublimation process with a 1 nm water layer on the sample surface [50]. Perhaps an easier method, although one that was not explored in this work, would be to stop the sublimation with a surface water layer between 10 – 100 nm, and then use cluster sputter etching (C_60_ or large argon clusters) to remove the remaining water to reach the plasma membrane. The sublimation step is not necessary for freeze-fracture experiments on single cells because a pristine intracellular surface is produced by the fracture process [18, 51]. Due to the difficulty of producing a uniform, thin ice layer, freeze-fracturing may be a better choice for the FH analysis of cells' plasma membranes.

Chemical fixation has caused the plasma membrane to become disorganized in some instances, but not in others. Recent work that imaged the sphingomyelin domains in NIH/3T3 cells used gluteraldehyde-fixation and observed that the plasma membrane was not disorganized [34]. A gluteraldehyde-fixed hTERT-BJ1 fibroblast cell did not show signs of membrane rupture in the m/z 184^+^ signal [24]. Cells that were fixed with formalin and freeze dried [33], as well as NIH/3T3 fibroblasts fixed with formaldehyde [52] did show evidence of plasma membrane rupture. Gluteraldehyde can cross-link the free amine group in phosphoserine and phosphoethanolamine, prominent components of the inner leaflet of the plasma membrane, while formaldehyde cannot. This may be the explanation for the disorganized plasma membranes seen when formaldehyde was used as the fixating agent.

### Comparing the depth profiles of FD and FF cells

One of the most exciting prospects of biological sample analysis with ToF-SIMS is depth profiling and the accurate 3D reconstruction of mass-spectral data sets [33, 52]. Low ion yields are the largest hurdle to overcome when acquiring 3D images [53], as there often isn’t enough signal intensity for most organic ions of interest to produce a meaningful reconstruction. Salts played a significant role in the suppression of SI yields obtained from depth profiles of rat brain tissue [21] and polypeptide films [20]. To test the extent to which positive SI intensities could be “recovered” by the removal of a majority of intracellular salts (Na^+^, K^+^, and Cl^-^), NIH/3T3 fibroblasts were prepared using two methods and depth profiles were acquired. In the first method, cells were plunge-frozen in liquid ethane and freeze dried, which preserved the native intracellular gradients of mobile species. In the second method, cells were fixed with formaldehyde, rinsed to remove buffer salts, and air dried. Chemical fixation will disrupt the native concentration gradients of mobile ions [24, 31], and as such, with heavy rinsing, can be used a as sample preparation method that produces single cells with minimal intracellular salts.

The possibility that intracellular molecules were removed during FF process was examined. The membrane is permeabilized to small molecules during the fixation procedure. Therefore, it is possible some of the intracellular molecules could pass through the permeabilized membrane and be removed during the rinsing step. However, all of the positive ions detected from the FD cells were also detected in the FF cells. Thus, for the instrument parameters and the preparation methodologies used in this study, no removal of intracellular organic ions was detected.

**Figure 4:**
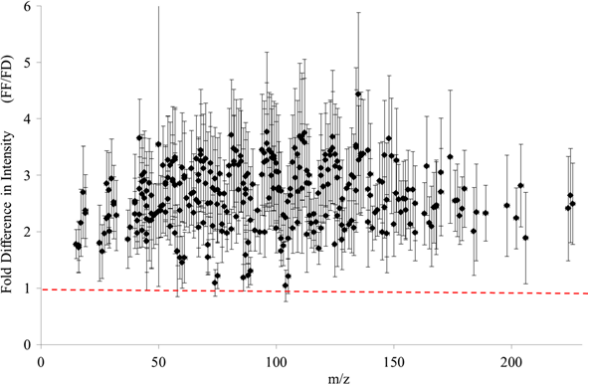
**Depth profiles were acquired from cells that were freeze-dried (FD) and from those that were formaldehyde-fixed (FF).** 311 ions were selected that were detected in the spectra of both sample types. The intensity of each ion was summed over the entire depth profile. Intensity values for each ion were averaged and normalized to the total ion dose. For a given ion, the average intensity from the FF cells was divided by the average intensity from the FD cells. The fold difference is plotted on the y-axis as a function of m/z. The red dotted line signifies a (FF/FD) intensity of 1. Error bars signify one standard deviation. All depth profiles were acquired for 1000 seconds, with a 500 μm × 500 μm analysis area and a 700 μm × 700 μm sputter area. For the FD cells, n = 10, from four total samples and three separate data acquisition sessions. For the FF cells, n = 7, from three total samples and three separate data acquisition sessions.

311 common ions were present in the spectra obtained from the two preparation methods. The fold difference in the average, normalized intensity (FF/FD) for each ion is shown in Figure 4. The horizontal red line at 1 indicates the same normalized intensity was collected for a given ion from both preparation methods. The normalized intensities of the selected SIs were higher from the depth profiles of FF cells compared to the FD cells. Of the 311 peaks, 14.6% of the ions had an increase in intensity of less than 2×, 56.3% increased between 2× and 3×, and 29.1% increased greater than 3x compared to the FD cells. For the entire peak set, the FF cells had an average increased intensity of 2.6 fold compared to the FD cells. The numerical values of the fold differences of all 311 SIs are shown in Additional file 3: Table S1. Two-tail student’s *t*-tests assuming unequal variance were conducted to determine if the intensity differences were statistically significant. The results are shown in Additional file 3: Table S1. Using a *p*-value threshold of 0.05, 309 of the 311 ions had statistically significant intensity differences. The two ions that did not exhibit a significant intensity difference were C_4_H_12_N^+^ and C_5_H_14_NO^+^ at m/z 74.10^+^ and 104.11^+^.

The intensities of the characteristic PC fragments at m/z 58^+^, 86^+^, 104^+^, 125^+^, and 184^+^[54, 55] were among the ions with the lowest fold increase. They had fold increases at or below 2.1 (color-coded yellow in Additional file 3: Table S1) compared to the 2.6 fold average increase, and include the ions with the lowest (m/z 104.11^+^) and third lowest increase (m/z 86.06^+^). The intensity of hydrocarbon fragments (color-coded blue) generally increased less than nitrogen- and oxygen-containing fragments. The SIs associated with proteins [56, 57], were often nitrogen-containing (color-coded green) and generally had a larger fold increase than the hydrocarbon and lipid-associated ions. SIs containing oxygen, but not nitrogen (color-coded red) did not display a clear trend, however they also generally had higher fold increase than the lipid and hydrocarbon ions. Despite the nitrogen and/or oxygen content, the fragments originating from PC did not exhibit a large increase. A similar finding was reported when the m/z 86^+^ SI intensity did not change appreciably in depth profiles of rat brain tissue that had been rinsed versus not rinsed, whereas the m/z 184 did have in increased intensity when the tissue was rinsed [21].

**Figure 5:**
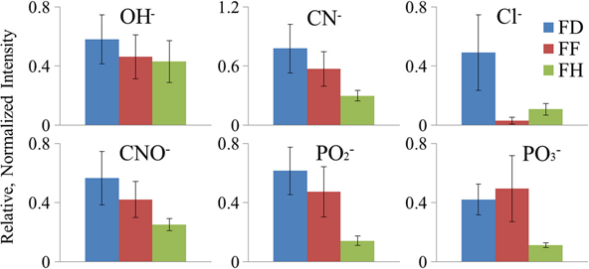
**Depth profiles were acquired from cells that were freeze-dried (FD, blue bars), formaldehyde-fixed (FF, red bars) and frozen-hydrated (FH, green bars).** The average, normalized intensity of the OH^-^, CN^-^, Cl^-^. CNO^-^, PO_2_^-^, and PO_3_^-^ ions were summed over the entire depth profile and plotted. Intensity values for each ion were normalized to the total ion dose. Error bars signify one standard deviation. All depth profiles were acquired for 1000 seconds, using a 500 μm × 500 μm analysis area and a 700 μm × 700 μm sputter area. For the FD cells, n = 10, from four total samples and three separate data acquisition sessions. For the FF cells, n = 7, from three total samples and three separate data acquisition sessions. For the FH cells, n = 11, from three total samples and three separate data acquisition sessions.

It was previously hypothesized that the neutralization of positive SIs by Cl^-^ anions in KCl-doped polypeptide films was the major mechanism of the lowered observed SI yields versus un-doped films [20]. The strongest piece of evidence that supported this mechanism over the others that were proposed was the decreased yield of *all* observed organic ions from the films in the presence of Cl^-^ containing salts. In this work, a similar decrease in all organic SI yields was observed in the FD cells compared to the FF cells. The relative, normalized SI signals from the OH^-^, CN^-^, Cl^-^, CNO^-^, PO_2_^-^ and PO_3_^-^ anions are plotted in Figure 5. These were examined to determine if they could explain the trend observed in Figure 4. The Cl^-^ anion had a statistically significant, higher SI intensity in the FD cells compared to the FF cells. The other five anions except Cl^-^ did not have a statically different SI intensity between the FD and FF cells, although all of the anions except PO_3_^-^ had higher mean intensities. This suggests that the anion neutralization mechanism hypothesized by Piwowar *et al*. is the underlying mechanism that caused the decreased SI intensities acquired from the FD cells, and that the removal of the intracellular Cl^-^ by fixation and rinsing can improve SI yields.

Depth profiles from freeze-dried and formaldehyde fixed cells are shown in Additional file 2: Figure S2 A and B, respectively, depicting the Poisson-corrected intensity of selected secondary ions as a function of C_60_^++^ sputter time. The depth profiles were normalized to the Bi_3_^+^ ion dose. The NH_4_^+^, Na^+^, K^+^, C_3_H_8_N^+^, C_4_H_8_N^+^, C_5_H_12_N^+^ and C_5_H_15_PNO_4_^+^ ions are displayed. For the organic molecules, the line shapes from the two depth profiles look qualitatively similar. In both cases, the initial intensities of the organic molecules were the highest, followed by a quick decay with increasing sputter dose. The normalized intensities of the organic molecules are also all higher throughout the depth profile of the formaldehyde-fixed cells, consistent with the data presented in Figure 4.

### Comparing the depth profiles of FD and FH cells

There was a larger difference between the summed depth profile spectra of the FH cells and the FD cells than between the FF and FD cells. It was more difficult to compare the spectra of the FH cells to the FD cells than the spectra of the FD to the FF. The mass resolution was, generally, worse in the spectra of FH cells than the dried cells, which made the selection of peaks present in both types of spectra difficult. One explanation is that the Bi_3_^+^ analysis source was optimally tuned in between analyses of the dry samples, whereas the Bi_3_^+^ source could only be tuned 2–3 hours before the analysis of the FH samples began, and not during the data acquisition. A second possible explanation focuses on the dehydration effect of cells. Full dehydration can collapse cells, reducing the height of dried cells compared to the hydrated form. The mass resolution obtained by the ION-TOF V instrument is sensitive to height variations, and since the hydrated cells were taller than the dried cells (AFM micrographs, data not shown), the mass resolution in the spectra of the FH cells may have been reduced compared to the flatter, dehydrated cells.

There were numerous peaks in the spectra from FH cells that were not detected, or detected in very low amounts, in the spectra of the FD cells. These ions are listed in Additional file 4: Table S2 with possible chemical identifications. No peak assignments indicate that a rational identification was not determined. A majority of these “new” peaks were assigned combinations of water, the ammonium acetate rinsing solution, and intracellular salt ions. In depth profiles of FH samples, water clusters such as H_3_O-H_2_O^+^ and H_3_O-(H_2_O)_2_^+^ at m/z 37 and 55 initially decreased in intensity and then increased with time (data not shown). The initial decrease was attributed to the removal of surface water, which adsorbed after the initial sublimation step. Water clusters with Na^+^ and K^+^ adducts were also detected, e.g. K-H_2_O^+^. Many of the ions detected in the FH but not FD spectra included NH_3_^+^ or NH_4_^+^ in their compositions, which can be attributed at least in part due to the AA rinsing solution. Examples include the ions detected at m/z 52.09^+^ and 53.07^+^, which were identified as (NH_3_)_2_-NH_4_^+^ and (NH_3_)_2_-H_3_O^+^. The intensity of the m/z 53.07^+^ fragment decreased rapidly initially, in a similar fashion to the other water clusters. Since not all of the ions could be identified with reasonable certainty, it is possible that some of them are more biologically relevant than simply being water- or salt-containing.

**Figure 6:**
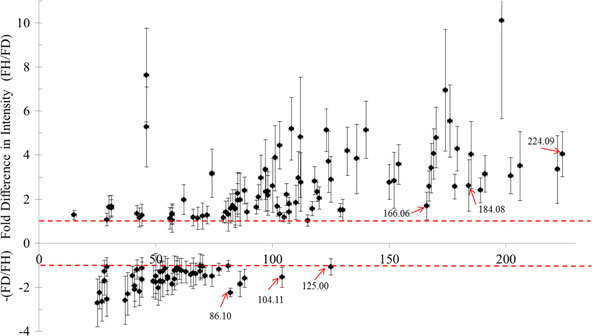
**Depth profiles were acquired from cells that were freeze-dried (FD) and from those that were frozen-hydrated (FH).** 137 ions were selected that were detected in the spectra of both sample types. The intensity of each ion was summed over the entire depth profile. Intensity values for each ion were averaged and normalized to the total ion dose. For a given ion, the average intensity from the FD cells was divided by the average intensity from the FH cells. If this ratio was below one, the -(FD/FH) ratio was used instead. The fold difference is plotted on the y-axis as a function of m/z. The red dotted lines signify (FH/FD) and (FD/FF) intensities of 1. Error bars signify one standard deviation. All depth profiles were acquired for 1000 seconds, with a 500 μm × 500 μm analysis area and a 700 μm × 700 μm sputter area. For the FD cells, n = 10, from four total samples and three separate data acquisition sessions. For the FH cells, n = 11, from three total samples and three separate data acquisition sessions.

The fold difference between the normalized intensities of the 137 SIs detected from both the FH and FD samples are shown in Figure 6. The SIs with a higher intensity from the FH cells were plotted in the top half of Figure 6 using a positive fold increase (FH/FD > 1), whereas the SIs with a higher intensity from the FD cells were plotted in the bottom half of Figure 6 using a negative fold increase (FH/FD < −1). The red, dashed lines at 1 and −1 indicate a ratio of 1, and thus no intensity difference. The numerical values of the fold difference of all ions are shown in Additional file 5: Table S3. Two-tail student’s *t*-tests were performed assuming unequal variance to determine if the intensity differences were statistically significant. A majority of SIs with m/z < 80 had higher intensities from the FD cells. The low mass SIs that had a statistically significant increased intensity in the FH spectra were CH_3_^+^, CH_4_N^+^, CH_3_O^+^, CH_5_N^+^, C_2_H_2_O^+^, CH_4_NO^+^, C_2_H_8_N^+^, C_3_H_7_N^+^, C_3_H_5_O^+^, and possibly C_3_H_3_Na^+^. All of these ions except CH_3_^+^ possibly contain an NH_4_^+^, H_2_O^+^ or H_3_O^+^ group. An increased SI intensity was obtained for larger masses in FH samples compared to the FD samples. An increase in the FH/FD ratio with increasing mass of the fragment was observed for the PC fragments at m/z 86^+^, 104^+^, 125^+^, 166^+^, 184^+^ and 224^+^ (labeled with red arrows in Figure 6). The m/z 86 fragment had the lowest fold difference (−1.9) for FD/FH. This value increased for the larger fragments at m/z 104^+^ and 125^+^, although the intensities from the FD cells were still higher than from the FH cells. Of the PC-related ions, the m/z 166 fragment had a SI yield higher in the FH cells, and the m/z 224^+^ had the greatest fold increase. In fact, all selected SIs above m/z 89^+^ except the m/z 104^+^ and 125^+^ (both PC fragments) had an increased intensity from the FH cells compared to the FD cells.

The SI yield results described above are in agreement with previous findings from cryogenic depth profiling experiments of polypeptide films. An increased [M + H^+^] intensity in the peptide films was observed when they were cooled from room to cryogenic temperatures during analysis, although there was little to no increase in lower molecular weight fragments [37]. The increased molecular ion signal was attributed to a decreased damage cross-section, produced by the presence of surface water and cryogenic temperatures. More recent work compared the spectra following C_60_ sputter etching from within chemically-fixed, freeze-dried HeLa-M cells to FH cells and observed higher yields for the characteristic DNA peaks at m/z 136^+^, 152^+^, as well as the PC peak at m/z 184^+^[33]. In that study case, lower mass peaks were not investigated. The results presented in Figure 6 from depth profiles of NIH/3T3 fibroblasts support the hypothesis that the primary mechanism of increased molecular SI signals result from a decreased damage cross section produced by cryogenic temperatures. The other mechanisms that have been proposed as a source of increased yields from FH analysis, namely proton donation [58] and amelioration of anion neutralization [20] by water, may also be playing a role, but from the data presented in Figure 6, it is unclear to what extent.

The accumulated intensities of particular anions from FH cells are shown in Figure 5 alongside the intensities from FD and FF cells. Well-preserved, hydrated cells should have a similar amount of these species as FD cells, yet the observed SI intensities were significantly lower (*p* < 0.05) for all but OH^-^. Incorporated water plays an important role in determining the yield of negatively charged monatomic species. Water ice drastically reduced the SI yields of negative ions compared to positive monatomic ions in computational studies [59, 60], and this may explain why the SI intensities were lower in the FH cells. The insignificant decrease in the OH^-^ intensity was not surprising as it is a water fragment. Protons donated by the intracellular water may also combine with the anions to form neutral, undetected species.

A representative depth profile from a sample with frozen-hydrated cells is shown in Additional file 2: Figure S2 C, depicting the Poisson-corrected intensity of selected secondary ions as a function of C_60_^++^ sputter time. H_2_O^+^ and H_5_O_2_^+^ profiles are shown in addition to the same fragment profiles shown in Additional file 2: Figure S2 A and B. As before, qualitatively the intensity profiles of the organic molecules are similar to those observed from the FD and FF cells. Although the initial normalized intensities are lower for all of the selected organic molecules, the intensity decay over time appears lower compared to the profiles in Additional file 2: Figure S2 A and B. This is consistent with a lowered damage cross section in the FH depth profile compared to the profiles of the dried cells. Unlike in Additional file 2: Figure S2 A and B, in Figure S2 C the PC headgroup (C_5_H_15_PNO_4_^+^) molecule had a higher initial intensity than the headgroup fragments (C_3_H_8_N^+^ and C_5_H_12_N^+^). The intensity of NH_4_^+^ starts and remains high throughout the profile, likely from the ammonium acetate rinsing solution.

Given the data presented above, it would be interesting to further investigate the secondary ion yield increases that may be obtained by first doing chemical fixation to remove diffusible salts ions, and then cryogenic analysis for decreased damage. This experiment would help determine the relative contributions of proton donation from water versus decreased damage from cryogenic temperatures.

## Conclusions

Sample preparation plays a key role in determining the information that is obtained from single cells with ToF-SIMS. The removal of excess media before analysis is necessary, but using rinsing technique that was too aggressive damaged the plasma membrane. The presence of intracellular salts reduced the secondary ion yield an average of 2.6-fold. Chemical fixation followed by rinsing removed a majority of the intracellular salts, “recovering” the positive secondary ion yields. Cl^-^ ion yields were highest in the freeze-dried cells and lowest in the formaldehyde-fixed cells. The data presented here is consistent with anion neutralization as the dominant mechanism for the lower ion yields. All of the organic secondary ions that were detected in the freeze-dried cells were also detected in the formaldehyde-fixed cells. Well-preserved, hydrated cells showed no increase or a decreased yield for most low mass ions, but an increased yield for higher mass fragments. This is consistent with the mechanism where a reduced damage cross section is produced by analysis at cryogenic temperatures. Numerous ions that were detected from the frozen-hydrated cells were not detected from the freeze-dried cells, however many of these ions were attributed to chemical combinations of water, salt and the ammonium acetate rinsing solution.

Future considerations regarding the optimal sample preparation for depth profiling cells would be need to be addressed in a case specific manner (e.g., for experiments that involve mapping small molecule drugs in cells). It may be beneficial (or necessary) to utilize chemical fixation or frozen-hydrated analysis to increase SI yields, but if the molecule is unbound and is removed or relocated during preparation, cryofixation would be necessary for accurate imaging.

## Electronic supplementary material

Additional file 1: Figure S1: (**A**) Na^+^ peak and **(B)**^41^ K^+^ peak from the depth profile of FH cells. The K^+^/Na^+^ ratio is 14.7, signifying the cells were well preserved. The ^41^ K^+^ isotope peak was used due to detector saturation of the ^39^ K^+^ isotope peak. (PDF 42 KB)

Additional file 2: Figure S2: **(A)** Depth profile from cells that were freeze-dried. **(B)** Depth profile from cells that were chemically fixed with formaldehyde. **(C)** Depth profile from cells that were analyzed frozen-hydrated. The depth profiles were normalized by the Bi_3_^+^ ion dose. (PDF 311 KB)

Additional file 3: Table S1: All positive secondary ions detected from both the FD and FF cells. Sorted by fold difference value, smallest to largest. (PDF 124 KB)

Additional file 4: Table S2: Positive Ions that were detected from the frozen-hydrated cells but not the freeze-dried cells, or in new very low amounts in the FD cells. (PDF 29 KB)

Additional file 5: Table S3: All positive secondary ions detected from both the FH cells and the FD cells. (PDF 52 KB)
