# Supplementary figures and images for: Characterization of sample preparation methods of NIH/3T3 fibroblasts for ToF-SIMS analysis
Source: Biointerphases. 2013 Jul 5;8(1):15. doi: 10.1186/1559-4106-8-15 (PMC4000548; doi:10.1186/1559-4106-8-15)

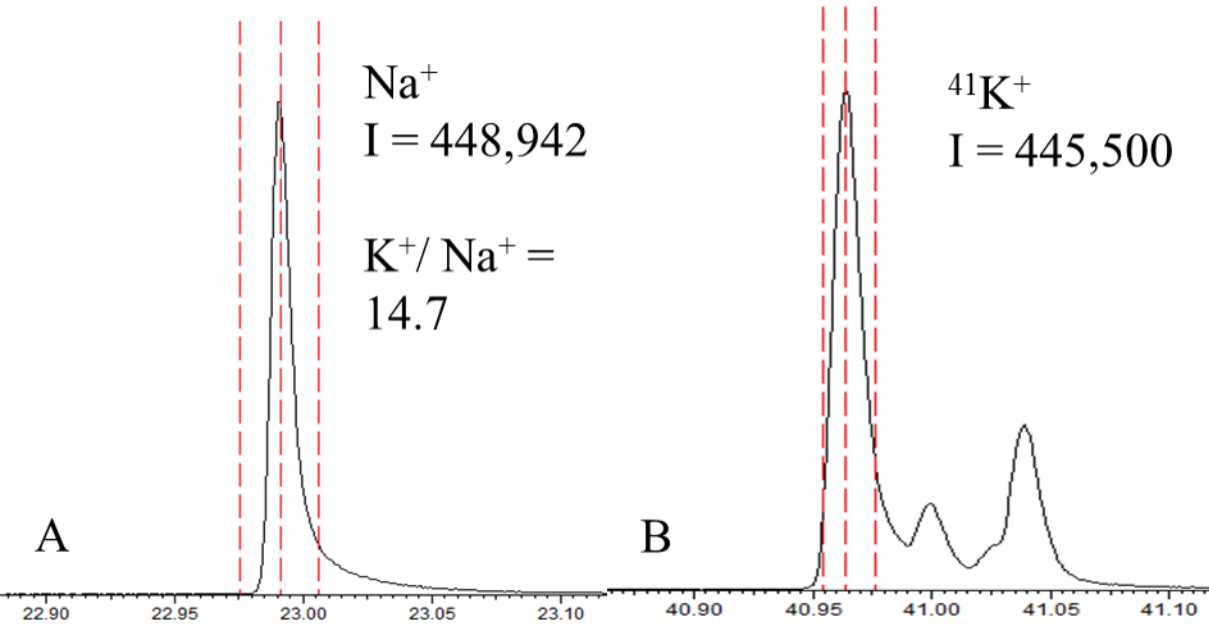

Supplement: Supplementary file 1 — Additional file 1: Figure S1: (A) Na+ peak and (B)41 K+ peak from the depth profile of FH cells. The K+/Na+ ratio is 14.7, signifying the cells were well preserved. The 41 K+ isotope peak was used due to detector saturation of the 39 K+ isotope peak. (PDF 42 KB) [file BJIOBN-000008-000015_1-s001.pdf]

Intensity (Normalized by ion dose)

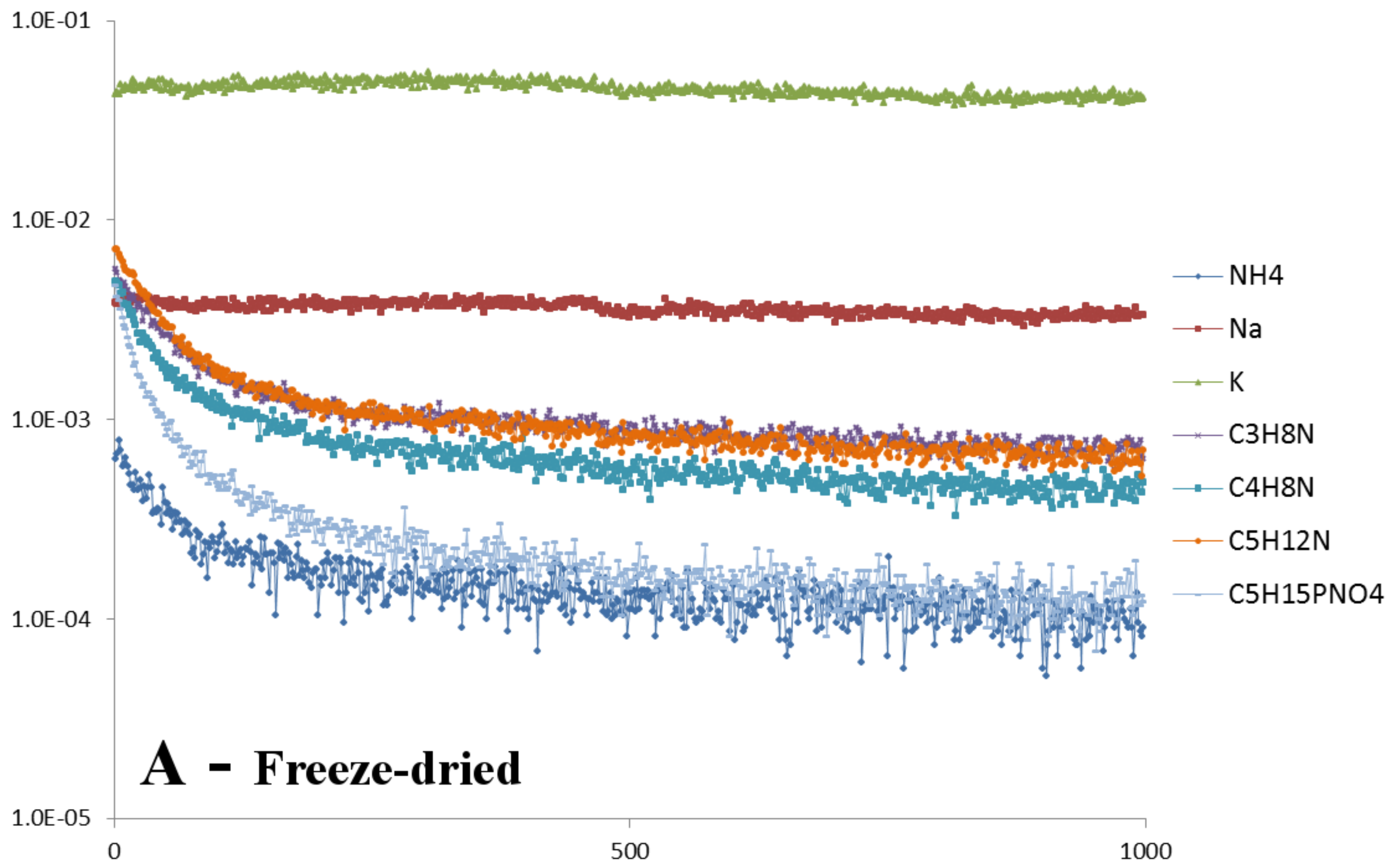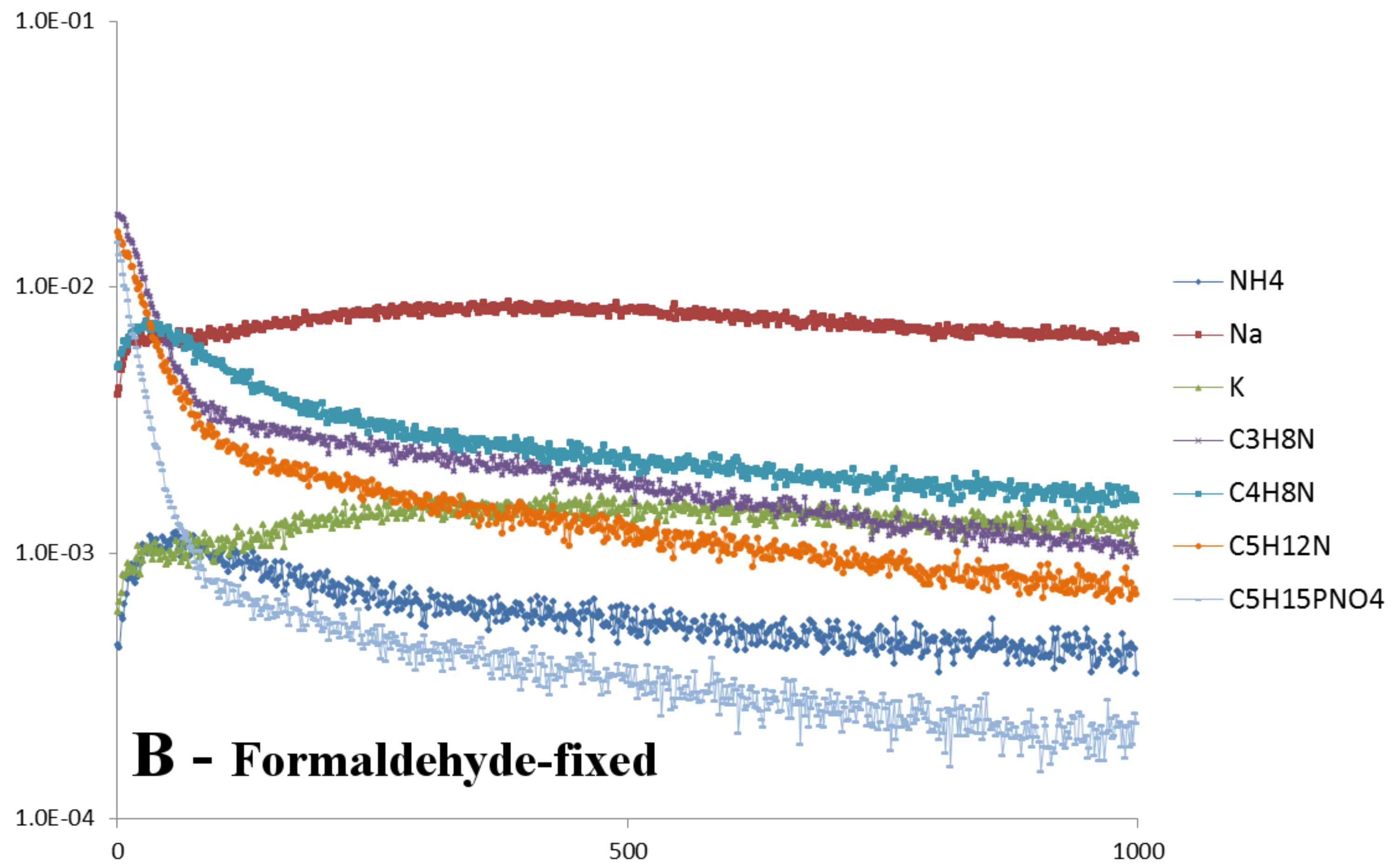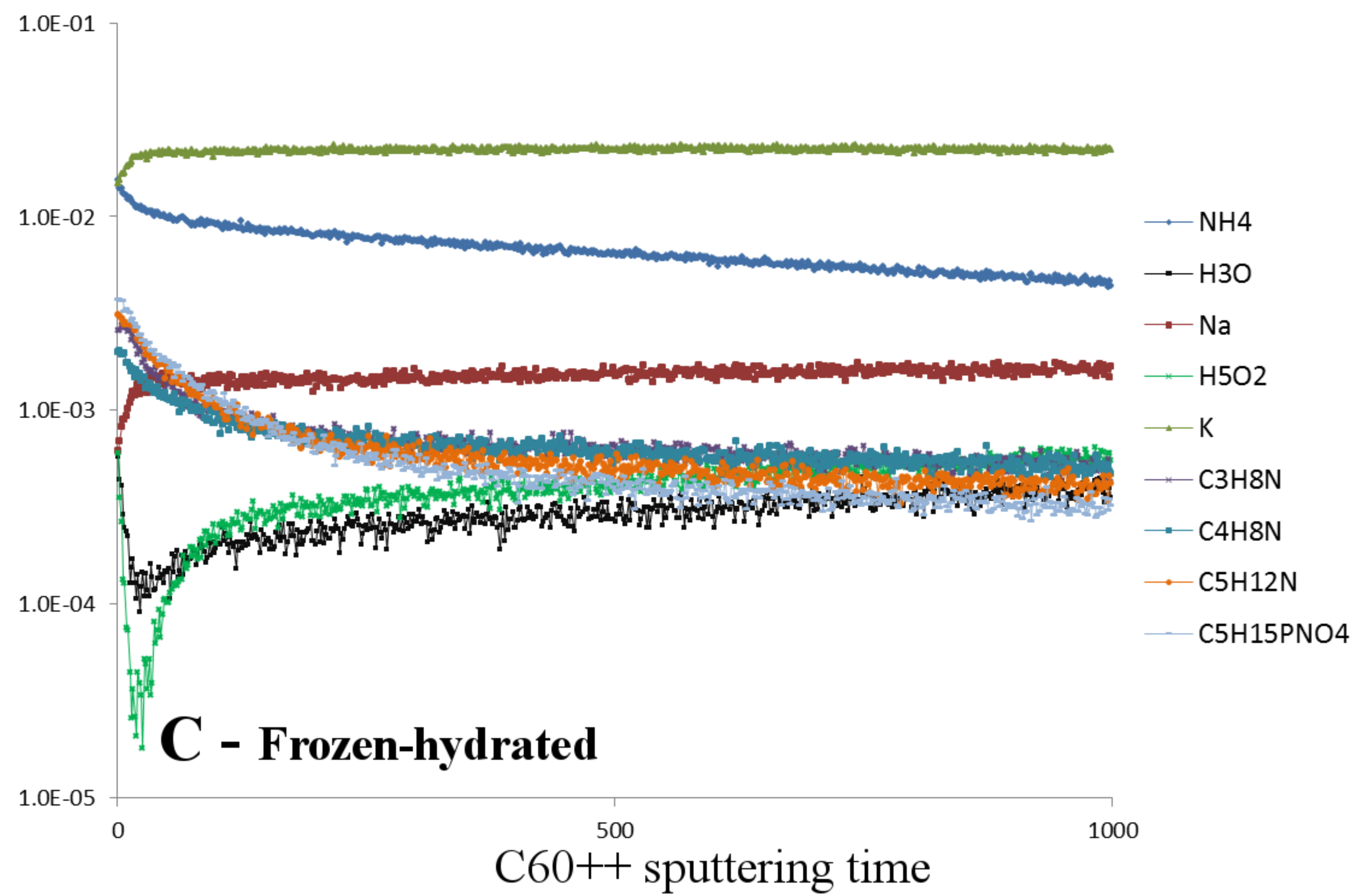

Supplement: Supplementary file 2 — Additional file 2: Figure S2: (A) Depth profile from cells that were freeze-dried. (B) Depth profile from cells that were chemically fixed with formaldehyde. (C) Depth profile from cells that were analyzed frozen-hydrated. The depth profiles were normalized by the Bi3+ ion dose. (PDF 311 KB) [file BJIOBN-000008-000015_1-s002.pdf]
