# Supplementary material for: Characterization of sample preparation methods of NIH/3T3 fibroblasts for ToF-SIMS analysis
Source: Biointerphases. 2013 Jul 5;8(1):15. doi: 10.1186/1559-4106-8-15 (PMC4000548; doi:10.1186/1559-4106-8-15)
Supplement: Supplementary file 3 — Additional file 3: Table S1: All positive secondary ions detected from both the FD and FF cells. Sorted by fold difference value, smallest to largest. (PDF 124 KB) [file BJIOBN-000008-000015_1-s003.pdf]

Table S.1: All positive secondary ions detected from both the FD and FF cells. Sorted by fold difference value, smallest to largest.

Mass	Fold difference	stdev	*t*- test	Possible composition
104.11	1.04	0.28	7.47E-01	C5H14NO
74.10	1.09	0.24	2.74E-01	C4H12N
86.10	1.18	0.24	3.08E-02	C5H12N
105.11	1.21	0.31	4.79E-02	isotope
75.10	1.22	0.23	9.20E-03	isotope
88.11	1.22	0.21	4.05E-03	C5H12N
89.11	1.30	0.25	1.45E-03	isotope
60.08	1.46	0.46	3.01E-03	
59.07	1.54	0.37	1.01E-04	
61.09	1.54	0.45	5.38E-04	
71.07	1.55	0.30	1.44E-05	
87.10	1.59	0.40	1.04E-04	
26.01	1.65	0.48	8.89E-05	
58.06	1.65	0.26	2.55E-08	C3H8N
102.09	1.66	0.28	1.59E-07	C5H12NO
58.03	1.66	0.82	4.99E-03	
118.12	1.70	0.28	3.28E-08	
16.03	1.72	0.44	5.14E-06	
16.02	1.73	0.46	3.91E-06	
103.09	1.75	0.36	2.28E-07	
16.03	1.76	0.27	1.98E-08	
71.04	1.77	0.49	1.85E-05	
125.00	1.78	0.58	4.07E-05	
15.02	1.78	0.26	1.17E-08	CH3
25.01	1.80	0.67	1.97E-04	
88.08	1.81	0.37	6.60E-08	
45.03	1.83	0.55	1.15E-05	
128.02	1.85	0.53	3.61E-06	
37.00	1.87	0.51	5.85E-06	
102.13	1.88	0.39	3.52E-08	
105.07	1.88	0.36	2.98E-05	C8H9
206.05	1.89	0.81	2.49E-04	
113.00	1.95	0.67	9.77E-04	
45.02	1.96	1.00	4.55E-04	
147.11	1.96	0.69	5.03E-05	
27.02	1.96	0.32	1.10E-10	C2H3
41.02	1.97	0.37	2.98E-08	
57.07	1.97	0.36	6.31E-06	C4H9
79.06	1.98	0.33	1.07E-08	
159.11	1.98	0.50	3.35E-04	
145.09	1.99	0.49	5.73E-04	
93.07	1.99	0.38	9.21E-06	
116.11	1.99	0.36	2.27E-10	
95.09	1.99	0.44	1.92E-04	

Phosphocholine
Hydrocarbon
Protein
$C_xH_yO_z$
Sulfur-containing

77.00	2.00	0.55	4.28E-07	
29.04	2.00	0.29	4.49E-10	C2H5
184.09	2.00	0.78	2.92E-05	C5H15NPO4
43.05	2.02	0.29	2.59E-08	C3H7
91.05	2.02	0.36	4.09E-08	C7H7
129.06	2.03	0.39	4.80E-07	
81.07	2.04	0.36	3.01E-06	
107.08	2.06	0.44	8.28E-05	
100.11	2.06	0.43	6.90E-09	
141.06	2.06	0.39	1.08E-07	
119.09	2.06	0.41	3.73E-05	
133.10	2.06	0.49	1.68E-04	
38.01	2.07	0.52	1.54E-07	
67.05	2.07	0.36	3.22E-09	C5H7
55.05	2.09	0.32	9.57E-10	C4H7
166.06	2.09	0.71	3.29E-06	C5H13NPO3
73.09	2.10	0.55	5.22E-08	
128.06	2.11	0.42	3.43E-08	
131.08	2.12	0.42	7.57E-06	
157.06	2.13	0.45	1.11E-08	
77.04	2.13	0.40	9.75E-10	C6H5
150.06	2.13	0.57	4.24E-07	C5H12SNO2?
115.05	2.15	0.43	2.02E-08	C4H7N2O2?
17.03	2.16	0.59	3.01E-08	NH3
165.05	2.16	0.46	3.33E-08	
69.07	2.16	0.39	7.77E-08	C5H9
109.10	2.16	0.52	1.51E-04	
139.01	2.17	0.80	5.75E-05	
117.06	2.17	0.42	2.62E-08	
41.04	2.19	0.37	5.14E-11	C3H5
46.07	2.19	0.71	3.69E-07	
132.08	2.19	0.57	8.77E-09	
89.08	2.20	0.54	3.79E-09	
47.04	2.20	0.84	6.26E-06	
45.05	2.23	0.52	2.81E-09	C2H7N
28.03	2.23	0.46	1.63E-10	C2H4
202.04	2.24	0.54	8.15E-08	
104.05	2.25	0.42	8.57E-10	
83.09	2.26	0.46	4.20E-07	
48.00	2.28	0.57	1.75E-08	
121.10	2.28	0.54	2.45E-05	
126.01	2.28	0.90	4.52E-05	
29.00	2.28	0.49	3.38E-10	
178.05	2.28	0.51	7.75E-08	
114.13	2.29	0.41	3.06E-11	
32.05	2.29	0.62	5.21E-09	
103.05	2.30	0.48	2.31E-09	
42.01	2.31	0.59	2.09E-08	
116.05	2.31	0.46	6.18E-09	

40.03	2.31	0.40	2.09E-11	
49.00	2.32	0.54	2.11E-09	
47.06	2.32	0.98	5.63E-06	
163.04	2.32	0.55	8.00E-08	
189.04	2.33	0.50	1.70E-08	
19.02	2.33	0.45	1.17E-08	
65.04	2.33	0.45	1.76E-10	
142.06	2.34	0.49	1.05E-08	
185.08	2.34	0.86	1.55E-06	
153.05	2.34	0.56	3.72E-08	
53.04	2.35	0.42	2.58E-10	C4H5
79.02	2.35	0.49	5.70E-10	
50.01	2.36	0.47	1.57E-10	
92.05	2.37	0.46	1.79E-10	C3H10SN
19.03	2.37	0.64	8.02E-09	
51.02	2.37	0.48	1.28E-10	C4H3
59.03	2.38	0.81	1.19E-07	
155.08	2.38	0.53	1.49E-06	
143.07	2.38	0.46	7.30E-08	C10H9N
179.06	2.41	0.57	5.05E-07	
127.05	2.41	0.55	9.32E-09	
224.10	2.41	0.93	1.56E-06	
167.06	2.43	0.63	3.89E-09	
146.09	2.43	0.54	4.15E-08	
168.03	2.44	0.84	7.35E-08	
78.05	2.44	0.46	1.52E-08	
51.01	2.45	0.55	8.89E-08	
44.99	2.45	0.73	1.34E-08	
198.09	2.46	0.89	8.50E-07	
63.02	2.46	0.48	7.60E-12	
168.07	2.47	0.77	1.42E-08	
52.03	2.47	0.50	4.44E-12	
31.02	2.49	0.48	2.33E-08	CH3O
75.02	2.49	0.69	1.27E-09	C3H7S
226.05	2.49	0.72	2.61E-09	
159.08	2.50	0.66	1.37E-08	
71.01	2.50	1.27	7.58E-06	C3H3O2
40.02	2.50	0.44	2.25E-11	
72.08	2.51	0.55	2.25E-10	
67.02	2.51	0.96	2.80E-07	
86.07	2.51	1.32	7.71E-06	
31.04	2.52	0.65	4.28E-10	CH5N
105.04	2.53	0.64	2.94E-08	
40.00	2.54	0.83	1.72E-05	
176.04	2.55	0.54	8.08E-08	
177.04	2.56	0.65	1.66E-07	
147.04	2.56	1.01	9.41E-07	
66.04	2.56	0.48	2.94E-11	
152.05	2.57	0.63	2.53E-08	

155.04	2.58	0.69	1.88E-08	
128.11	2.58	0.69	1.44E-10	
54.04	2.58	0.49	6.37E-10	
89.03	2.59	0.57	4.74E-10	
154.05	2.59	0.66	7.66E-09	
97.10	2.59	0.55	9.29E-08	
62.01	2.59	0.51	1.55E-12	
44.03	2.60	0.71	2.45E-08	CH4N2
47.01	2.64	0.78	1.30E-07	
106.06	2.64	0.56	7.04E-10	
225.10	2.64	0.84	6.42E-09	
140.04	2.64	0.65	1.35E-07	
87.02	2.66	0.55	6.95E-11	
43.02	2.67	0.56	2.74E-07	C2H3O
76.03	2.67	0.64	9.10E-10	
78.04	2.67	0.59	7.01E-12	
64.03	2.68	0.56	4.10E-10	
118.07	2.68	0.58	5.98E-10	
151.04	2.68	0.69	6.56E-08	
18.04	2.70	0.82	6.11E-09	NH4
65.01	2.70	0.69	4.03E-08	
170.09	2.70	0.77	1.51E-08	
109.22	2.71	0.61	2.82E-09	
102.04	2.72	0.70	1.98E-09	
45.04	2.72	0.70	2.81E-10	C2H5O
134.10	2.73	0.74	1.48E-07	
74.02	2.73	0.72	3.05E-09	
101.03	2.73	0.68	3.36E-09	
29.03	2.74	0.67	4.25E-09	
75.07	2.74	0.93	5.94E-09	
158.08	2.74	0.68	1.86E-09	
84.08	2.75	0.79	3.67E-10	C5H10N
156.07	2.75	0.70	1.90E-09	
139.04	2.75	0.68	1.18E-07	
87.08	2.75	0.88	1.16E-08	
180.06	2.76	0.68	1.05E-09	
106.03	2.76	0.89	4.98E-09	
42.04	2.76	0.56	6.68E-10	
111.01	2.76	1.14	2.78E-03	
130.06	2.76	0.71	1.57E-10	C9H8N
99.02	2.77	0.75	3.13E-09	
73.07	2.78	0.91	3.03E-09	C2H7N3 or C3H7NO
101.10	2.78	0.81	7.66E-10	
120.05	2.80	0.63	6.40E-10	
120.08	2.80	0.65	1.73E-09	C8H10N
204.06	2.82	0.74	2.21E-09	
57.03	2.82	0.72	9.25E-08	
57.05	2.83	0.57	3.18E-12	C3H7N
131.04	2.84	0.66	6.58E-10	C9H7O

53.02	2.84	0.66	6.30E-09	
122.10	2.84	0.63	3.22E-09	
90.04	2.84	0.63	2.02E-12	
28.02	2.85	0.59	4.76E-10	CH2N
70.07	2.85	0.76	2.56E-10	C4H8N
59.05	2.85	0.75	5.74E-10	CH5N3 or C3H7O or C2H5NO
86.01	2.85	0.62	1.54E-12	
114.05	2.86	0.83	4.56E-09	
46.03	2.86	0.78	1.98E-09	
113.03	2.87	0.69	1.46E-08	
53.00	2.87	0.66	6.55E-11	C3HO
145.06	2.88	0.72	1.40E-08	
43.04	2.89	0.59	9.10E-13	C2H5N
56.05	2.90	0.68	8.21E-11	C3H6N
67.03	2.90	0.74	1.36E-09	
144.07	2.90	0.67	1.23E-09	
44.05	2.91	0.75	7.23E-11	C2H6N
56.02	2.92	0.78	1.93E-09	
30.03	2.94	0.70	2.65E-10	CH4N
74.07	2.94	0.90	4.61E-09	C3H8NO
61.01	2.95	0.80	1.89E-11	C2H5S
54.00	2.95	1.10	3.31E-08	
108.08	2.96	0.71	6.47E-11	
126.05	2.96	0.76	1.51E-08	
133.07	2.97	0.76	5.11E-11	
87.06	2.98	0.95	3.10E-09	C3H7N2O
61.05	2.99	0.97	1.88E-09	C2H7NO
98.01	2.99	0.90	8.34E-06	
119.06	3.00	0.71	1.75E-08	
80.05	3.00	0.69	2.07E-10	
114.09	3.00	0.82	1.86E-10	
132.05	3.00	0.83	6.78E-11	
43.03	3.01	0.71	2.45E-09	CH3N2
94.06	3.01	0.72	1.89E-10	
67.04	3.01	0.66	4.10E-10	
140.10	3.02	0.90	1.53E-10	
76.02	3.03	0.72	1.48E-12	
88.04	3.03	0.74	2.23E-10	C3H6NO2
44.01	3.05	0.74	2.42E-10	CH2NO
170.05	3.05	0.96	1.94E-09	
101.07	3.06	1.01	2.62E-08	C4H9N2O
72.04	3.06	0.80	1.55E-09	C3H6NO
100.08	3.07	0.88	6.07E-10	
113.08	3.08	0.82	7.00E-10	
122.06	3.10	0.81	2.39E-10	
64.01	3.12	0.67	1.28E-12	
69.06	3.12	0.78	2.51E-11	C4H7N
127.10	3.13	0.90	2.60E-10	
119.03	3.13	0.96	1.99E-07	

60.04	3.14	0.97	6.54E-08	C2H6NO
110.07	3.14	0.99	1.78E-09	C5H8N3
60.05	3.14	0.96	2.09E-08	CH56N3
125.07	3.16	0.92	1.87E-11	
164.04	3.16	0.88	3.09E-07	
80.02	3.17	1.02	6.64E-09	
55.02	3.18	0.77	1.88E-09	C3H3O
52.01	3.18	0.69	2.84E-12	
84.05	3.19	1.00	1.47E-09	
82.05	3.19	0.86	8.96E-11	C4H6N2
100.04	3.21	1.06	1.38E-09	
72.06	3.22	0.89	4.09E-09	
95.02	3.23	0.95	2.58E-10	
107.05	3.23	0.84	1.07E-09	C7H7O
56.01	3.24	0.97	2.70E-08	
126.09	3.25	0.94	3.00E-10	
85.08	3.25	0.90	1.21E-11	
82.07	3.25	0.81	1.16E-10	
69.03	3.25	0.83	2.05E-09	C4H5O
68.05	3.25	0.81	1.13E-10	C4H6N
151.09	3.26	0.92	2.10E-11	
96.08	3.27	0.81	6.21E-10	
121.08	3.27	0.83	2.13E-10	
135.06	3.27	0.96	2.24E-10	
54.03	3.27	0.77	5.87E-11	C3H4N
57.02	3.28	0.92	5.12E-09	
99.09	3.29	0.93	3.25E-11	
70.03	3.30	0.98	4.19E-09	C3H4NO
66.03	3.30	0.76	4.19E-12	
98.09	3.31	0.86	6.36E-11	C6H12N
57.04	3.32	0.85	1.99E-10	
174.02	3.32	1.18	3.14E-08	
99.05	3.34	0.97	6.41E-10	
136.05	3.34	1.01	2.09E-07	
149.06	3.34	1.02	1.93E-10	
85.04	3.34	1.01	5.35E-09	C3H5N2O
123.06	3.36	0.98	5.40E-11	
146.05	3.36	0.97	2.40E-11	
68.02	3.37	0.90	1.46E-09	
121.04	3.37	0.91	1.38E-10	C6H5N2O
109.07	3.37	0.95	9.76E-11	
125.11	3.37	0.89	6.05E-12	
136.07	3.38	1.09	1.18E-08	C8H10NO
137.07	3.38	0.96	6.73E-10	
95.06	3.39	0.93	7.75E-10	C5H7N2
98.06	3.40	1.04	7.90E-10	C5H8NO
123.09	3.43	0.88	1.06E-10	
83.06	3.43	0.91	4.24E-10	C5H7O
68.01	3.44	1.08	1.81E-08	

97.07	3.44	0.94	1.65E-09	C6H9O
139.08	3.45	1.06	4.37E-10	
97.03	3.45	1.02	1.03E-08	
94.03	3.45	0.99	3.95E-11	
82.03	3.48	1.04	8.78E-09	C4H4NO
108.05	3.48	1.06	7.84E-10	
124.08	3.49	1.00	4.53E-10	
134.06	3.50	1.01	2.10E-10	
134.02	3.53	1.37	1.68E-07	
50.00	3.55	2.51	2.77E-03	
96.04	3.56	1.07	1.84E-09	
112.09	3.58	1.14	2.61E-11	
111.05	3.63	1.07	2.75E-09	
148.06	3.65	1.12	1.10E-08	
42.03	3.66	0.69	6.71E-15	C2H4N
124.04	3.68	1.18	9.22E-08	
110.02	3.69	1.30	3.66E-06	
111.09	3.69	1.13	4.37E-10	
81.04	3.71	0.98	8.75E-08	C4H5N2?
112.04	3.75	1.31	2.63E-07	DNA C4H6N3O
96.01	3.77	1.41	1.42E-05	
135.02	4.44	1.45	1.39E-06	
