# Supplementary material for: Characterization of sample preparation methods of NIH/3T3 fibroblasts for ToF-SIMS analysis
Source: Biointerphases. 2013 Jul 5;8(1):15. doi: 10.1186/1559-4106-8-15 (PMC4000548; doi:10.1186/1559-4106-8-15)
Supplement: Supplementary file 4 — Additional file 4: Table S2: Positive Ions that were detected from the frozen-hydrated cells but not the freeze-dried cells, or in new very low amounts in the FD cells. (PDF 29 KB) [file BJIOBN-000008-000015_1-s004.pdf]

Table S.2: Positive Ions that were detected from the frozen-hydrated cells but not the freeze-dried cells, or in new very low amounts in the FD cells.

M/z	Composition	Possible structure
18.04	NH_4	NH_4
19.02	H_3O	H_3O
19.03		
19.04		
32.05	C_2H_6N	NH_4-CH_2
33.02	NH_3O	H_3O-N
33.03	CH_5O	H_3O-CH_2
33.05	N_2H_5	NH_3-NH_2
34.03	NH_4O	H_3O-NH
34.05	N_2H_6	NH_3-NH_3
35.03	NH_5O	H_3O-NH_2
35.06	N_2H_7	NH_4-NH_3
36.04	NH_6O	NH_3-H_3O
37.02	H_5O_2	H_3O-H_2O
43.02^	C2H3O	C2H3O
45.05"	CH5N2	(NH2)2-CH
47.06	CH_7N_2	$(NH_3)_2$-CH
48.04	CH_6NO	NH_3-H_2O-CH
49.08	CH_9N_2	$(NH_3)_2$-CH_3
50.06	CH_8NO	NH_4-H_2O-CH_2
52.09	N_3H_{10}	$(NH_3)_2$-NH_4
53.07	N_2H_9O	$(NH_3)_2$-H_3O
54.05	NH_8O_2	NH_3-H_5O_2
55.03	H_7O_3	H_3O-$(H_2O)_2$
55.99	KNH_3	K-NH_3
56.97	KH_2O	K-H_2O
59.01	NaH_4O_2	Na-$(H_2O)_2$
59.07^"	$C_2H_7N_2$	$(NH_3)_2$-C_2H
60.05^"	C_2H_6NO	NH_3-C_2H_3O
61.03^	$C_2H_5O_2$	$C_2H_5O_2$
61.08"	$C_2H_9N_2$	$(NH_3)_2$-C_2H_3
62.06	C_2H_8NO	NH_4-C_2H_3O-H
63.05	CH_7N_2O	$(NH_3)_2$-CHO
73.05	H_9O_4	H_3O-$(H_2O)_3$
74.01^"		
74.99	KH_4O_2	K-$(H_2O)_2$
75.06		
76.05		
76.08		
77.02	NaH_6O_3	Na-$(H_2O)_3$
77.08	CN_4H_9	$(NH_3)_3$-CN

^ Possible NH4-acetate fragment

" overlaps different peaks in dried cells

78.01		
78.06^	$C_2H_9NO_2$	NH_4-$C_2H_4O_2$
79.00		
80.00		
80.99		
83.01^	$NaC_2H_5O_2$	Na-$C_2H_5O_2$
83.99		
88.05		
89.07	$C_3H_9N_2O$	$(NH_3)_2$-C_3H_3O
90.07	$NH_{12}O_4$	NH_3-H_9O_4
92.02		
92.08^	$C_3H_{10}NO_2$	NH_4-$C_2H_5O_2$-CH
92.99	KH_3O_6	K-$(H_2O)_3$
96.07		
98.00	C_2H_5NOK	K-NH2-C2H3O
98.99	$KC_2H_4O_2$	K-$C_2H_4O_2$
99.99	$KC_2H_5O_2$	K-$C_2H_5O_2$
100.04	$C_4H_6NO_2$	C_2H_2N-$C_2H_5O_2$
101.03		
102.00		
102.06		
109.06	$H_{13}O_6$	H_3O-$(H_2O)_5$
111.00	KH_8O_4	K-$(H_2O)_4$
114.00		
114.08		
116.02		
117.00		
126.01		
126.10	$NH_{16}O_6$	NH_3-$H_{13}O_6$
127.07	$H_{15}O_7$	H_3O-$(H_2O)_6$
129.01	$KH_{10}O_5$	K-$(H_2O)_5$
137.09		
138.09		
143.03		
144.10	$NH_{18}O_7$	NH_3-$H_{15}O_7$
145.07	$H_{17}O_8$	H_3O-$(H_2O)_7$
147.02	$KH_{12}O_6$	K-$(H_2O)_6$
148.06		
152.94		
155.10		
158.03		
159.01		
160.01		
160.06		
161.00		
162.09		
163.08	$H_{19}O_9$	H_3O-$(H_2O)_8$

173.03		
174.09		
180.98		
181.09	$H_{21}O_{10}$	$H_3O\text{-}(H_2O)_9$
194.96		
195.98		
196.96		
197.08		
199.10	$H_{23}O_{11}$	$H_3O\text{-}(H_2O)_{10}$
