# Supplementary material for: Characterization of sample preparation methods of NIH/3T3 fibroblasts for ToF-SIMS analysis
Source: Biointerphases. 2013 Jul 5;8(1):15. doi: 10.1186/1559-4106-8-15 (PMC4000548; doi:10.1186/1559-4106-8-15)
Supplement: Supplementary file 5 — Additional file 5: Table S3: All positive secondary ions detected from both the FH cells and the FD cells. (PDF 52 KB) [file BJIOBN-000008-000015_1-s005.pdf]

Table S.3: All positive secondary ions detected from both the FH cells and the FD cells

Mass	Fold Difference	Stdev	*t*- test	Mass	Fold Difference	Stdev	*t*- test
15.02	1.28	0.22	1.04E-04	60.08	-1.18	0.48	2.12E-01
25.01	-2.72	1.09	4.05E-04	61.01	-1.24	0.56	1.21E-01
26.01	-2.25	0.74	1.41E-04	62.01	1.98	0.68	1.69E-04
27.02	-2.66	0.88	1.38E-08	63.02	-1.31	0.49	2.46E-02
28.02	-1.29	0.51	3.99E-02	65.04	-1.44	0.53	2.76E-03
28.03	-1.71	0.65	1.35E-04	66.03	-1.36	0.70	4.50E-02
29.00	1.07	0.29	3.92E-01	66.04	1.18	0.38	1.24E-01
29.03	-1.08	0.42	5.32E-01	67.05	-1.40	0.49	3.47E-03
29.04	-2.54	0.79	1.45E-09	68.05	1.13	0.52	3.96E-01
30.03	1.65	0.52	1.05E-04	69.04	-1.04	0.55	8.23E-01
31.02	1.61	0.38	4.87E-06	69.07	-1.28	0.40	1.21E-02
31.04	1.68	0.50	1.46E-05	70.03	1.24	0.59	1.76E-01
37.00	-2.60	1.09	3.04E-05	70.07	-1.07	0.53	6.77E-01
38.01	-2.30	0.96	1.65E-05	71.07	-1.49	0.40	3.06E-04
40.03	-1.50	0.43	1.61E-04	72.09	1.28	0.40	2.11E-02
41.02	-1.93	0.71	2.92E-06	74.07	3.17	1.08	1.81E-08
41.04	-2.11	0.79	4.13E-07	74.10	-1.50	0.47	1.25E-03
42.01	1.35	0.37	1.45E-03	77.04	-1.20	0.28	2.66E-02
42.03	-1.21	0.44	9.51E-02	79.06	1.16	0.23	2.01E-02
43.04	1.17	0.43	2.11E-01	80.05	1.42	0.50	6.69E-03
43.05	-2.19	0.65	3.82E-09	81.04	1.30	0.76	1.95E-01
44.01	-1.14	0.48	3.29E-01	81.07	-1.04	0.25	6.19E-01
44.03	-1.66	0.80	1.60E-03	82.03	-2.24	0.20	1.48E-04
44.05	1.28	0.49	5.95E-02	82.07	1.59	0.66	5.45E-03
46.03	7.62	2.13	1.40E-12	83.06	1.72	0.72	1.94E-03
46.06	5.27	1.82	2.94E-11	84.05	1.54	0.71	1.14E-02
49.00	-1.73	0.72	2.93E-04	84.09	1.58	0.86	3.04E-02
50.00	-1.51	1.04	1.22E-01	85.04	1.96	0.76	7.94E-05
50.01	-1.77	0.52	2.88E-05	85.08	2.26	0.94	1.20E-04
51.02	-2.02	0.81	8.20E-06	86.07	1.98	1.20	1.07E-03
52.01	-1.74	0.86	6.46E-04	86.10	-1.86	0.58	1.08E-05
52.03	-1.31	0.50	2.52E-02	88.08	2.41	0.61	1.07E-08
53.00	-1.29	0.47	3.13E-02	88.11	-1.60	0.40	3.42E-05
53.04	-1.75	0.69	4.31E-05	89.03	1.43	0.41	7.72E-04
54.03	-1.15	0.54	3.24E-01	93.07	1.64	0.31	2.83E-08
55.02	-1.52	0.83	1.16E-02	94.06	2.10	0.56	9.69E-09
55.05	-1.62	0.53	3.02E-05	95.09	2.98	1.00	2.98E-05
56.05	1.13	0.48	3.64E-01	97.04	3.34	1.34	4.67E-06
57.02	1.05	0.55	7.57E-01	97.07	2.34	0.88	2.07E-05
57.03	1.30	0.44	2.34E-02	98.06	2.18	0.91	8.76E-05
57.05	1.34	0.45	1.68E-02	98.10	2.36	0.87	1.25E-05
57.07	-1.86	0.49	1.64E-07	100.08	2.61	1.22	2.66E-04
58.03	-1.25	0.67	2.43E-01	101.08	3.86	1.48	2.65E-07
58.06	-1.64	0.48	1.02E-05	102.09	1.67	0.44	3.04E-05
59.05	-1.24	0.61	1.61E-01	103.05	1.32	0.31	8.14E-04
59.07	-1.11	0.31	2.50E-01	103.09	4.43	1.08	2.82E-10

Mass	Fold Difference	Stdev	*t*- test
104.11	-1.55	0.46	1.80E-03
105.07	1.18	0.21	6.86E-03
106.06	2.23	0.50	6.39E-11
107.05	1.41	0.51	7.19E-03
107.08	1.78	0.30	5.21E-09
108.08	5.18	1.43	7.99E-10
110.08	1.85	0.79	4.84E-04
111.06	2.98	1.17	4.22E-06
112.05	4.81	2.72	1.97E-04
112.08	2.78	1.32	1.12E-04
115.05	1.03	0.26	6.90E-01
117.06	1.58	0.32	3.71E-07
118.07	2.83	0.62	3.43E-14
119.09	2.35	0.44	5.49E-09
120.08	2.06	0.51	3.43E-09
123.09	5.12	0.98	3.76E-13
124.08	3.69	1.38	5.04E-12
125.00	-1.08	0.37	4.99E-01
125.07	2.90	1.03	2.29E-07
129.06	1.50	0.31	3.05E-06
130.06	1.52	0.48	4.22E-04
132.08	4.18	1.08	2.34E-15
136.07	3.82	1.57	4.19E-06
140.05	5.13	1.31	4.17E-10
150.06	2.78	0.78	4.87E-12
152.05	2.85	1.26	1.81E-04
154.05	3.57	0.89	1.17E-15
166.06	1.70	0.63	5.53E-05
167.06	2.60	0.68	6.30E-12
168.07	3.40	1.11	1.23E-12
169.07	4.06	1.13	8.93E-16
170.05	4.78	1.37	1.48E-16
174.02	6.94	2.77	7.62E-08
176.03	5.53	1.65	1.75E-07
178.05	2.58	0.56	2.01E-12
179.07	4.28	1.01	4.77E-11
184.08	2.62	1.16	8.37E-07
185.08	4.01	1.51	1.33E-13
189.04	2.41	0.57	9.72E-10
191.06	3.14	0.81	1.80E-10
198.10	10.10	4.46	2.72E-07
202.04	3.06	0.81	1.25E-09
206.05	3.50	1.56	3.53E-11
222.02	3.34	1.53	1.59E-06
224.10	4.03	1.01	3.06E-10
